# Isolated intestinal Ganglioneuromatosis: case report and literature review

**DOI:** 10.1186/s13052-021-01024-5

**Published:** 2021-03-30

**Authors:** Angela Mauro, Letizia Zenzeri, Francesco Esposito, Giovanni Gaglione, Caterina Strisciuglio, Emanuela Pilozzi, Vito Domenico Corleto, Chiara Ziparo, Giovanni Di Nardo

**Affiliations:** 1 Pediatric Emergency Unit, AORN Santobono-Pausilipon, Naples, Italy; 2 Department of Emergency Radiology, AORN Santobono-Pausilipon, Naples, Italy; 3 Pediatric Surgery Unit, AORN Santobono-Pausilipon, Naples, Italy; 4 Department of Woman, Child and General and Specialistic Surgery, University of Campania “Luigi Vanvitelli”, Naples, Italy; 5 Department of Clinical and Molecular Medicine, UOC Anatomia Patologica, Sant’ Andrea Hospital, University “La Sapienza”, Rome, Italy; 6 Department of Medical-Surgical Sciences and Translational Medicine, Sant’Andrea Hospital, Sapienza University of Rome, Rome, Italy; 7 NESMOS Department, Faculty of Medicine and Psychology, Sapienza University of Rome, Pediatric Unit, Sant’Andrea University Hospital, Rome, Italy

**Keywords:** Intestinal ganglioneuromatosis, Polyps, Abdominal pain, Case report, Pediatric

## Abstract

**Background:**

Intestinal Ganglioneuromatosis (IG) is a rare disorder of the enteric nervous system. In pediatric age it is often associated with genetic syndromes such as Neurofibromatosis 1 (NF1), multiple endocrine neoplasia type 2B (MEN2B) and Cowden syndrome (PTEN mutation), and ganglioneuromas (GNs) may be sometimes the first sign of the disease. Isolated GNs are rare and sporadic. Clinical symptom vary and depend on the size and on the location of the GNs. This disorder affects intestinal motility and it, consequently, causes changes in bowel habits, abdominal pain, occlusive symptoms and rarely lower gastrointestinal bleeding secondary to ulceration of the intestinal mucosa. On the other hand, patients can remain asymptomatic for many years.

**Case presentation:**

We describe a 9-year-old boy referred to our emergency department for right lower quadrant abdominal pain. No familial history for gastrointestinal disorders. No history of fever or weight loss. At physical examination, he had diffused abdominal pain. Abdominal ultrasonography showed a hypoechoic formation measuring 41.8 mm by 35 mm in the right lower quadrant of the abdomen. Routine blood tests were normal, but fecal occult blood test was positive. Abdominal TC confirmed the hypodense formation, of about 5 cm in transverse diameter, in the right hypochondrium that apparently invaginated in the caecum-last ileal loop. Colonoscopy showed in the cecum an invaginated polypoid lesion of the terminal ileal loop. Laparoscopic resection of the polypoid lesion was performed. Histological diagnosis of the large neoplasm observed in the terminal ileum was diffuse ganglioneuromatosis. NF1, RET and PTEN gene tests resulted negative for specific mutations. At the 1 year follow-up, the patient presented good general condition and blood tests, fecal occult blood test, esophagogastroduodenoscopy, colonoscopy and MR-enterography were negative.

**Conclusions:**

Only few cases are reported in literature of IG in pediatric age. Although rare, the present case suggests that this disorder must be taken in consideration in every patient with GI symptoms such as abdominal pain, constipation, lower intestinal bleeding, in order to avoid a delayed diagnosis.

**Supplementary Information:**

The online version contains supplementary material available at 10.1186/s13052-021-01024-5.

## Background

Intestinal Ganglioneuromatosis (IG) is a rare disorder of the enteric nervous system. Ganglioneuromas (GNs) are hamartomatous tumors that originate in sympathetic ganglia and adrenal glands. More rarely, they occur in the gastrointestinal tract, originating in enteric nervous system cells [1]. GNs are divided into three groups: Polypoid GN, Ganglioneuromatous Polyposis and diffuse ganglioneuromatosis and they can be mucosal or transmural [2]. The transmural diffuse form is strongly associated with genetic syndromes such as Neurofibromatosis 1 (NF1), multiple endocrine neoplasia type 2B (MEN2B) and Cowden syndrome (PTEN mutation), and it can sometimes be the first sign of the disease. Indeed, isolated GNs are rare and not usually associated with genetic disorders [3]. We report a rare case of isolated ileal GN in a 9-year-old boy.

## Case presentation

**Fig. 1:**
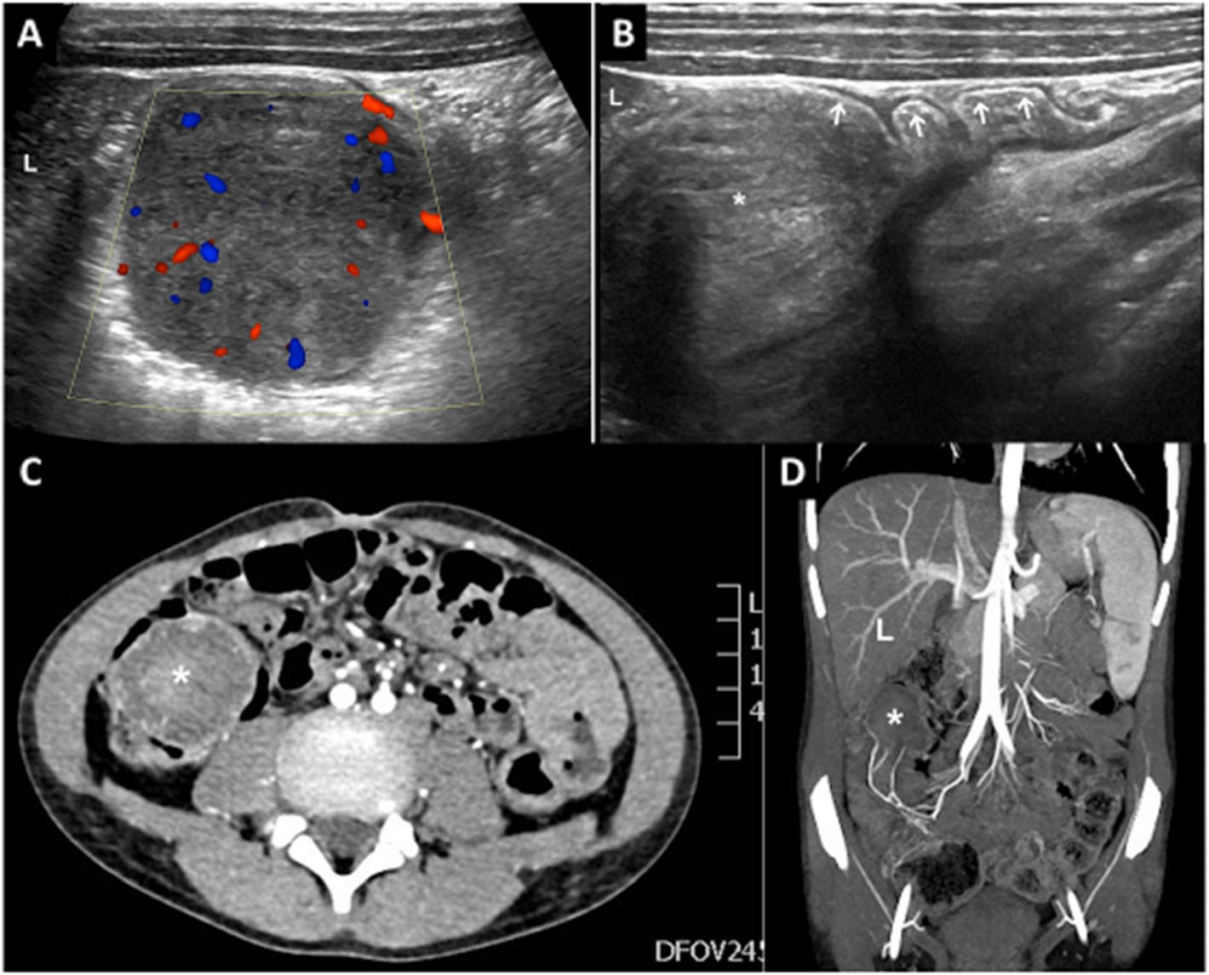
US Axial scan (**a**) shows a solid rounding mass in subheptical seat with intralesional vascularization; US longitudinal scan (**b**) shows a rounded mass (*) in the lumen of the colon (arrows); CT contrast exam, axial (**c**) and coronal (**d**) scan, show rounding solid mass (*) in subhepatic site. L = Liver

A 9-year-old boy was referred to our emergency department for acute and invalidant right lower quadrant abdominal pain. Family and physiological history were unremarkable. Because of the presence of lower gastrointestinal bleeding at 6 months of age, he started dietetic therapy with extensively hydrolyzed formula with good results until 18 months when he started a free diet. He had no history of fever, poor appetite, weight loss or constipation and diarrhea. Physical examination and vital parameters were normal, no lymphadenopathy or organomegaly. His abdomen was tender but he presented diffuse pain on abdominal palpation. For this reason, an abdominal ultrasonography was performed and it showed, in the right lower quadrant of the abdomen, edema of mesenteric tissues and of the last ileal loop, which was collapsed. Moreover, it revealed, an hypoechoic cecal polypoid lesion (41.8 mm by 35 mm) with intralesional vascularization (Fig. 1 a and b).

He was admitted to the pediatric gastroenterology unit where performed blood tests (red blood cell, white blood cell, hemoglobin level, platelet counts, electrolytes, alanine and aspartate transaminases, total and conjugate bilirubin, serum creatinine, urea, triglycerides, LDL and HDL cholesterol, blood glucose, inflammatory markers and all were in normal range), fecal occult blood tests (positive for blood), fecal calprotectin (200 μg/g) and an abdomen TC and colonoscopy were performed.

The abdomen TC revealed an hypodense formation of the last ileal loop, of about 5 cm in transverse diameter, in the right hypochondrium that apparently invaginated in the caecum. This formation presented inhomogeneous enhancement after contrast medium, highlighting numerous vascular poles. Inhomogeneous thickening of the contiguous loose cells was associated. The CT finding appeared suggestive of an expansive polypoid-like lesion on an invaginated loop (Fig. 1 c and d).

**Fig. 2:**
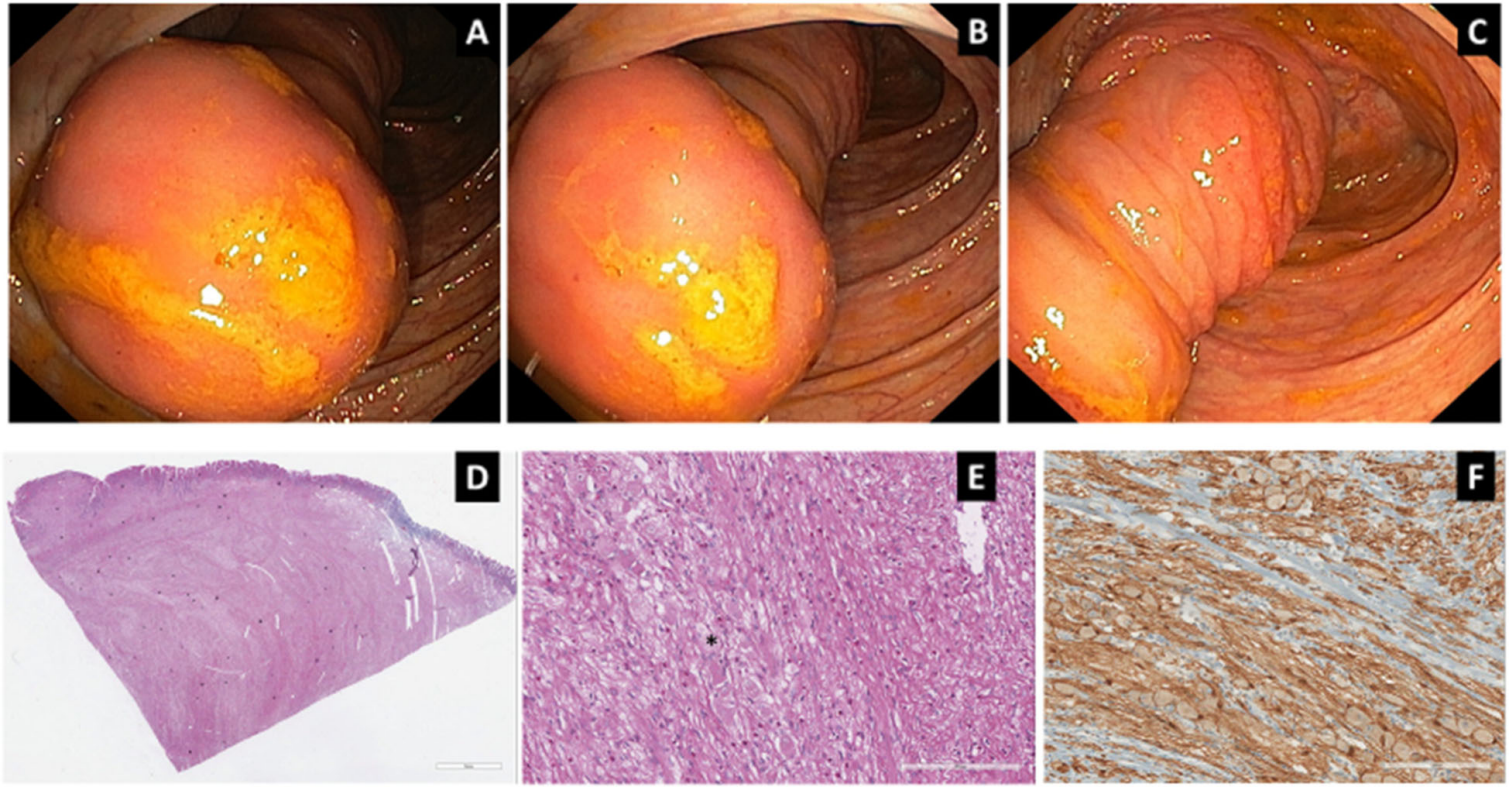
**a,b,c**) Colonoscopy showed a pedunculated polyp of the terminal ileum prolapsing in to the caecum. **d**) Section of ileum wall showing a transmural nodular proliferation of mature ganglion and spindle cells effacing all layers of intestinal wall (hematoxylin-eosin 2X). **e**) Prolifearation of spindle cells mixed with mature of ganglion cells (asterisk) (hematoxylin-eosin 20X). **f**) Strong positivity of ganglion and spindle cells for S-100 (Immunohistochemestry 20X)

Colonoscopy showed in the cecum an invaginated polypoid lesion of the terminal ileal loop (Fig. 2 a, b, c). Laparoscopy with resection of the polypoid lesion was performed. Macroscopic examination showed a large neoplasm in the terminal ileum wall, partially involving the ileocecal valve.

Microscopic examination revealed a transmural vaguely nodular proliferation, poorly demarcated, made of confluent mature ganglion cells and spindle cells with schwannian features. No mitosis were observed. Immunohistochemistry showed a strong staining of the ganglion cells for GFAP and S-100 and of spindle cells for S100. The neoplastic proliferation affected all layers from mucosa to muscularis propria effacing intestinal wall architecture. In the mucosa it caused expansion of lamina propria among crypts and was associated with focal pseudopyloric metaplasia. There wasn’t ulceration. Interspersed among neoplastic cells there were several eosinophils. (Fig. 2 d and e). According to WHO 2019 a diagnosis of diffuse ganglioneuromatosis was made.

Specific systemic disorders associated with diffuse ganglioneuromatosis presenting characteristic clinical stigmata such as, mucosal neuromas and marfanoid habitus in MEN2B, cafe’-au-lait spots and cutaneous neurofibromas in NF1, or trichilemmomas and “cobblestone” tongue lesions as in Cowden syndrome, were absent in the present case. However, we referred the patient for genetic analysis of NF1, RET and PTEN genes and all resulted negative for specific mutations. At the last follow-up after one year, the patient appeared in good general condition. General physical examinations, blood tests, fecal occult blood test, esophagogastroduodenoscopy, colonoscopy and MR-enterography were negative.

## Discussion

Intestinal GNs is a rare form of benign neurogenic tumor characterized by hyperplasia of ganglion cell nerve fibers and supporting cells [4].

According to WHO 2019 classification, intestinal ganglioneuroma can be broadly divided in 3 groups: solitary ganglioneuroma, a single mucosal polyp < 1–2 cm; ganglioneuromatous polyposis and diffuse ganglioneuromatosis. When presenting as a polyp, ganglioneuroma may histologically be reminiscent of juvenile polyp for cystic dilatation of glands.

In this context, an accurate differential diagnosis appears fundamental. About the 70% of colonic polyps is represented by juvenile solitary polyps, not related to malignant transformation.

This are reported meanly in children between 2 and 5 year of age with incidence from 0.08 to 3.7% [5, 6]. On the other end multiple polyps, rare conditions, are most suggestive of syndromic disease: hamartomatous syndrome as Juvenile polyposis syndrome, Bannayan-Riley-Ruvalcaba syndrome, Cowden’s syndrome or Beuz-Jeghers syndrome and adenomatous syndrome as Familian adenomatous polyposis, Turcot syndrome and Gardener sybdrome, typically associated with positive family history [7].

Polypoid GNs are usually adenomatous or hyperplastic small (< 2) juvenile tumors and can be either sessile or pedunculated in their morphology. Polypoid GNs can be solitary or come in small groups.

In ganglioneuromatous polyposis, there are usually a large number of polyps – groups of 20 and above are common – which can be sessile or pedunculated. They range from 1 mm to 2.2 cm in size and are found in the mucosa and/or submucosa. Such polyps can be filiform projections containing ganglion cells, or they can be impossible to distinguish from polypoid GNs in histological terms, and they have been linked to cutaneous lipomas. The diffuse ganglioneuromatosis lesions can present tissue that is nodular and either mucosal or transmural, involving the mesenteric plexus. Such lesions can be much larger, reaching sizes of 17 cm. They present as projections or proliferations; either fusiform hyperplastic projections, or irregular transmural proliferations that merge with the myenteric plexus [8].

Clinically, symptoms of GNs depend on the type of lesion, size and location. Polypoid GNs are typically asymptomatic or may present with abdominal pain, bleeding, obstruction, ileus, and constipation. Since the first report of GNs in 2000, only a few cases have been reported in the medical literature. Herein, we describe the clinicopathological features of this case for further understanding of the disease and review past cases in the literature (Table 1).

**Table 1:** Case reports of paediatric ganglioneuromatosis not associated to gene mutation

Authors	Age (sex)	Presentation	Location	Comorbidity	Diagnostic tool	Treatment
Soccorso et al. 2008 [9]	5 (girl)	Abdominal pain, bilious vomiting, bloody stool	colon	None	Ultrasound	Surgery (resection)
Matthews et al. 2013 [10]	7 (boy)	Abdominal pain, nausea	colon	Congenital neutropenia and growth hormone deficiency	Histological findings after surgery	Surgery (right hemicolectomy)
Mitra et al. 2016 [11]	11 (boy)	Abdominal distension, vomiting, constipation	Small intestine	None	Histological findings after surgery	Surgery (resection)

Indeed, Abraham et al. reported a case of a solitary colonic ganglioneuroma which presented with self-limited episodes of hematochezia due to the presence, on colonoscopy, of a 0.6 cm sessile polyp in the cecum, that produced a histological finding of ganglioneuroma [12].

Moreover, Soccorso et al. described a case of a 5 year-old-girl, who referred to the emergency department, as in our case, with abdominal pain, bilious vomiting and bloody stools caused by colonic intussusception. This diagnosis was made with US scan and she recived immediately laparotomic surgical treatment. A GN centered in the muscularis propria was identified [9]. Matthews et al. in the same way described a diffuse ganglioneuromatosis in 7-years-old male diagnosed after a surgical right hemicolectomy. In this case the child arrived to the emergency department with abdominal pain, diarreea, hematemesis and right-sided abdominal fullness. For the diagnosis, the authors performed acute abdominal series and CT-scan of the abdomen. After this the GNs was diagnose after surgical resection with surgical right hemicolectomy [10] (Table 1).

Recently, another case of 11-year-old boy with isolated ileal ganglioneuromatosis has been described. His clinical symptoms were abdominal distension, vomiting, and severe constipation. The diagnosis required necessary histopathological findings such as an extensive proliferation of ganglion cells in the submucosa with numerous hypertrophied nerve bundles extended to the serosa. In this case surgical treatment has been chosen because it is well known that endoscopic treatment is often inconclusive [11] (Table 1).

On the other side, MEN2B, NF1 and Cowden’s syndromes typically occur, associated with ganglioneuromatous polyposis and diffuse ganglioneuromatosis, rather than in association with isolated GNs.

Gfroerer et al. described a case of an infant with intestinal ganglioneuromatosis associated with MEN2B and reviewed the literature focusing on gastrointestinal symptoms of MEN2B. The main symptom was severe constipation, leading to a colonoscopy. A rectal biopsy was performed, and a precocious diagnosis of GNs and subsequently of MEN2B syndrome at 6 months of age was made [13]..

Nguyen et al., reported a case of isolated intestinal GN in association with MEN2B syndrome. In detail, the patient, at 10 years of age, presented melena and iron deficiency anemia that led to colonoscopy, which revealed three juvenile polyps that were removed, with subsequent improvement in the anemia. After 15 months, on a follow-up, colonoscopy showed a sessile polyp in the transverse colon, that presented histological characteristics of GN. Because of the presence of GN, the patient underwent a screening for MEN, that revealed a new polymorphism of the RET proto-oncogene [14].

Furthermore, Thay et al. described in a patient suffering from NF1 a case of intestinal diffuse GN accidentally found during surgery to remove multiple malignant peripheral nerve sheath tumors arising in the retroperitoneum [15].

Severe constipation has been described as predominant symptom also in a 6-years -old patient with diffuse ganglioneuromatosis associated to PTEN mutation. In this case it has been hypothesized that the GNs could be due to an altered proliferation of neural crest cells during embryonic growth of the enteric nervous system [16].

Moreover, concerning the association between Cowden’s syndrome and GNs, Herranz Bachiller et al. reported a case of diffuse colonic ganglioneuromas which presented with rectal bleeding associated with esophagogastric polyposis and facial trichilemmomas. Genetic tests for MEN2B and PTEN were performed, that resulted negative for the former and pending for the latter. Clinical diagnosis of Cowden’s syndrome was made and treatment started, with close follow-up in order to prevent any incidence of the malignancy related to this disease [17, 18].

Because GNs do not have endoscopic morphologically peculiar features, their diagnosis is usually made by microscopic and immunohistochemical examination of the biopsy that reveals immunoreactivity to S100 with the “comma-shaped” nuclei associated with aggregates of ganglion cells [19, 20].

Moreover, in our case, abdominal ultrasound (US) has been a crucial examination because it showed immediately the presence of the polypoid lesion. In literature, various studies underline the importance of abdominal ultrasound in the possible detection of polypoid lesions localized in the colon if they present a diameter greater than 2 cm. Because children present small body habitus and less fat tissue in the abdominal wall and peritoneal cavity, US could be a potential useful tool in the diagnostic work-up for abdominal pain associated to alarm signs such as bleeding, obstruction and ileus [21]. In addition, in order to rule out other GI disorders, clinician should be performed specific laboratory examinations as red blood cell, white blood cell, hemoglobin level, platelet counts, electrolytes, alanine and aspartate transaminases, total and conjugate bilirubin, triglycerides, blood glucose, iron balance, inflammatory markers, fecal occult blood tests and fecal calprotectin.

US cannot replace endoscopy in the diagnosis of colonic polyps, Moreover, this non-invasive instrumental examination could be useful also in patients with positive faecal tests, as in our case, because it could detect invaginated polypoid lesions that bleed because they are ulcerated [22]..

This approach might be a good compromise in patient with moderate alarm signs olso in terms of cost/benefit perspective.

Nowadays, no specific guidelines are available for treating GNs, and treatment of intestinal GNs depends on their size, location and associated symptoms. In the literature, some authors reported that solitary GNs were successfully treated by endoscopic complete resection [12, 14, 20, 23–25].

Literature data about GNs in pediatric population are scarce. It is for this reason, our patient performed a follow-up with esophagogastroduodenoscopy, colonoscopy and MR-enterography but because GNs are a benign lesion with excellent prognosis, a follow-up with colonoscopy i unnecessary. In fact, Shekitka et al. reported a 8-year follow-up study of 28 patients with solitary GNs during which none of the patients developed NF1 disease, MEN2B, recurrence of GNs or other complications [26].

## Conclusion

In conclusion, in every patient with recurrent abdominal pain, not due to functional gastro-intestinal disorder, although rare, the diagnosis of GNs need to be considered. In presence of alarm signs an abdominal US could help to identify polypoids lesions located in the colon. So, appropriate laboratory examination followed by an abdominal US, could be the first steps in making an early diagnosis and performing a medical/surgical treatment of intestinal GNs.

## Supplementary Information

**Additional file 1.**

## Data Availability

Data sharing is not applicable to this article as no datasets were generated or analyzed during the current study.

## Abbreviations

IG: Intestinal Ganglioneuromatosis
GNs: Ganglioneuromas
NF1: Neurofibromatosis 1
MEN2B: multiple endocrine neoplasia type 2B
NGS: next generation sequencing
US: ultrasound
