# Supplementary material for: Isolated intestinal Ganglioneuromatosis: case report and literature review
Source: Ital J Pediatr. 2021 Mar 30;47:80. doi: 10.1186/s13052-021-01024-5 (PMC8008650; doi:10.1186/s13052-021-01024-5)
Supplement: Supplementary file 1 — Additional file 1. [file 13052_2021_1024_MOESM1_ESM.docx]

| **Dates Relevant Past Medical History and Interventions** | | | |
| --- | --- | --- | --- |
|  | A 9-year-old boy was referred to our emergency department for acute and invalidant right lower quadrant abdominal pain. Family and physiological history were unremarkable. Because of the presence of lower gastrointestinal bleeding at 6 months of age, he started dietetic therapy with extensively hydrolyzed formula with good results until 18 months when he started a free diet. He had no history of fever, poor appetite, weight loss or constipation and diarrhea. | | |
| **Date** | **Summaries from Initial and Follow-up Visits** | **Diagnostic Testing (including dates)** | **Interventions** |
|  | Acute and invalidant right lower quadrant abdominal pain | Red blood cell, white blood cell, hemoglobin level, platelet counts, electrolytes, alanine and aspartate transaminases, total and conjugate bilirubin, serum creatinine, urea, triglycerides, LDL and HDL cholesterol, blood glucose, inflammatory markers and all were in normal range), fecal occult blood tests (positive for blood), fecal calprotectin (200 ug/g). | Admitted to the pediatric gastroenterology |
|  | Persisting abdominal pain | Abdominal ultrasonography  TC abdomen  Colonoscopy | Laparoscopy with resection of the polypoid lesion |
|  | Resection of the lesion | Histopathological examination | Diagnosis of intestinal ganglioneuromatosis |
